# Plant Leaf Position Estimation with Computer Vision

**DOI:** 10.3390/s20205933

**Published:** 2020-10-20

**Authors:** James Beadle, C. James Taylor, Kirsti Ashworth, David Cheneler

**Affiliations:** 1 Engineering Department, Lancaster University, Lancaster LA1 4YW, UK; j.beadle@lancaster.ac.uk (J.B.); d.cheneler@lancaster.ac.uk (D.C.); 2 Lancaster Environment Centre, Lancaster University, Lancaster LA1 4YW, UK; k.s.ashworth1@lancaster.ac.uk

**Keywords:** neural network, computer vision, depth estimation, position estimation, parallax

## Abstract

Autonomous analysis of plants, such as for phenotyping and health monitoring etc., often requires the reliable identification and localization of single leaves, a task complicated by their complex and variable shape. Robotic sensor platforms commonly use depth sensors that rely on either infrared light or ultrasound, in addition to imaging. However, infrared methods have the disadvantage of being affected by the presence of ambient light, and ultrasound methods generally have too wide a field of view, making them ineffective for measuring complex and intricate structures. Alternatives may include stereoscopic or structured light scanners, but these can be costly and overly complex to implement. This article presents a fully computer-vision based solution capable of estimating the three-dimensional location of all leaves of a subject plant with the use of a single digital camera autonomously positioned by a three-axis linear robot. A custom trained neural network was used to classify leaves captured in multiple images taken of a subject plant. Parallax calculations were applied to predict leaf depth, and from this, the three-dimensional position. This article demonstrates proof of concept of the method, and initial tests with positioned leaves suggest an expected error of 20 mm. Future modifications are identified to further improve accuracy and utility across different plant canopies.

## 1. Introduction

Fast phenotyping research facilities enable the rapid scanning of multiple plants for quality, growth, architecture, resource use efficiency and response to biological and environmental stresses [1,2]. Knowledge of these traits allows crop breeders to select cultivars best suited to particular growth conditions, improving potential yields [3].

Stresses such as herbivory, pathogenic infection, drought, extreme temperature and nutrient deficiency induce changes in plant gas exchange, including the emission of volatile organic compounds (VOCs) associated with biochemical changes in plant cells. VOCs can be used to detect early symptoms of stress, i.e., before there are visible changes, to enable timely (and therefore minimized) intervention. This is currently carried out in the field at the whole-crop scale using remote sensors but could also be applied for indoor facilities at the single plant or even individual leaf-level to enable greater monitoring precision. In this context, an autonomous system able to accurately position a portable chemical sensor, e.g., an “e-nose” [4,5], on a leaf surface or at a specified height above an individual plant would enable more rapid, accurate monitoring of individual plant stress status and reduce the costs associated with what is currently a labour-intensive process.

Recent activity in agricultural robotics has also investigated the automation of fast phenotyping for genetics as well as other purposes [6]. Photographic scanning is beneficial for monitoring small flowering plants such as Arabidopsis thaliana used in [6], however, leaf-level phenotyping of larger plants such as Vigna unguiculate (Cowpea), which was used in this study, can be used to monitor localized chemical traits.

A key issue in the implementation of an autonomous system capable of positioning a chemical sensor against individual leaves is with the estimation of leaf location. To predict the location of a leaf in three-dimensional space, it must first be located within a digital image. This can be achieved with convolutional neural networks, which are widely used for object detection-based tasks.

The use of neural networks in agriculture has been proven to be beneficial for easily gathering data. For example, identifying the current phenological stage of a crop [7] or, on a more localised level, phenotyping an individual plant [8]. Neural networks have commonly been used for locating specific plant features in images, such as leaves or fruit [9,10], which in turn allows for additional data to be gathered, e.g., the ripeness of fruit [11], or the detection of diseases and pests [12,13].

In addition to detecting objects in images, a depth measurement technique must also be applied. Current infrared (IR)-based depth mapping devices, for example, the Microsoft Kinect, use an IR point mesh and camera to create an RGB-D image. The accuracy of depth measurements taken by these devices is negatively affected by the presence of incandescent or ambient light, which washes out the IR point mesh [14,15]. Whether in a plantation or laboratory setting, this makes the use of these devices impractical. Time-of-flight depth measurement technologies, such as the Adafruit VL53L0X (LIDAR) and Multicomp HC-SR04 (SONAR), are commonly only capable of measuring the depth of a single point, instead of generating a depth map. Both methods require the device to be directed towards a point of interest and have a wide field of view of around 25° to 30° [16,17]. Due to these disadvantages, neither option is appropriate for measuring the depth of densely packed vegetation.

Block-matching algorithms used for depth mapping use only stereo image data to predict depth. Similar images taken at different locations are divided into subsections, and similar areas are matched [18]. The discrepancy in the position of a match is subsequently used to predict the relative depth. This technique has been used in devices such as the PlayStation camera, and in software packages such as OpenCV. Work by [19] demonstrates how this method can be used in agriculture, in the situation of locating and autonomously picking ripe tomatoes. Block-matching algorithms are best suited to mapping the depth of large objects and are less effective when scanning intricate scenes such as foliage. The small surface area of each leaf, combined with the fact most leaves are similar in colour and size, poses a challenge for algorithms designed to generate depth maps from stereo images [20]. This issue is addressed by [20], who designed a new block-matching algorithm which took an input of five images instead of only two. [21] also presents a novel approach, based on extracting the curvature of narrow-leaf plants such as wheat or barley, disregarding texture and colour.

Current state-of-the-art work includes [22], who propose a semi-automatic technique for modelling plants from images. The method produces highly accurate results for intricate plant architectures but requires 35 to 45 images to be taken of the subject plant from many different positions and angles, as well as human intervention to add in details missed by the imaging technique and algorithms.

The approach developed by [23] relies on bounding box detections for plant leaf depth estimation. The method relies on a recursive algorithm which first predicts the location of a target leaf. The camera is subsequently moved to a position that aims to increase estimation accuracy and the process is repeated. The results show promise; however, the method is only capable of estimating the position of a single leaf, meaning again many different camera positions and angles would be required to model a full plant.

The present article proposes an alternative approach to predicting the position of multiple wide leaves in three-dimensional space with a straightforward and easy-to-implement scanning procedure, and a computational approach to estimating depth and position that is not sensitive to dense vegetation, or to the presence of natural or incandescent light. Common block-matching algorithms attempt to estimate the depth of all locations in an image, while only position data related to each leaf needs to be considered. Removing all unnecessary information from the input image allows the applied grouping algorithm to be comparably simpler. The proposed method uses only input data from a digital camera, a computationally light-weight object detection neural network, a straightforward grouping algorithm, and the principle of parallax. Although the method was not tested in a real-world setting, the research demonstrates proof of concept and the future aim is for trials in field conditions. The proposed approach is described in Section 2. The neural network’s effectiveness is evaluated using live plants, and the proposed position estimation technique is evaluated using printed leaves in Section 3. Conclusions and suggestions for further development are presented in Section 4.

## 2. Materials and Methods

### 2.1. Robotic Platform Description

**Figure 1:**
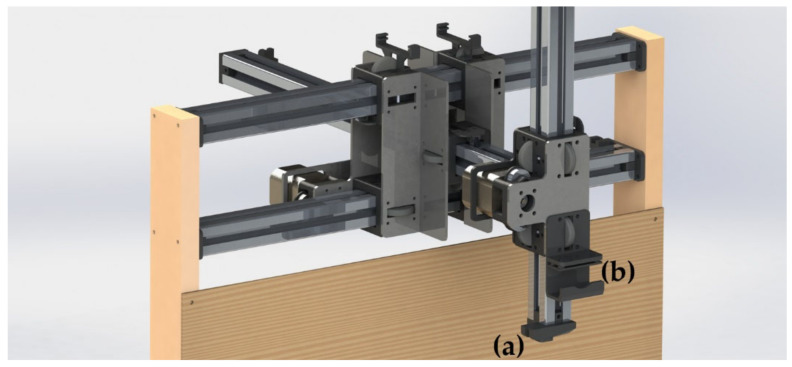
CAD model of the robotic platform showing the full mechanical layout used for three-dimensional positioning of the sensor located at point (**a**). Controllable motion in each axis allowed for the probing arm (**a**) to be positioned anywhere within a 250 mm × 250 mm × 330 mm volume. The PlayStation Eye camera was clamped in place at (**b**), facing downwards. The camera had a fixed height and could be moved horizontally within a 250 mm × 250 mm area.

**Figure 2:**
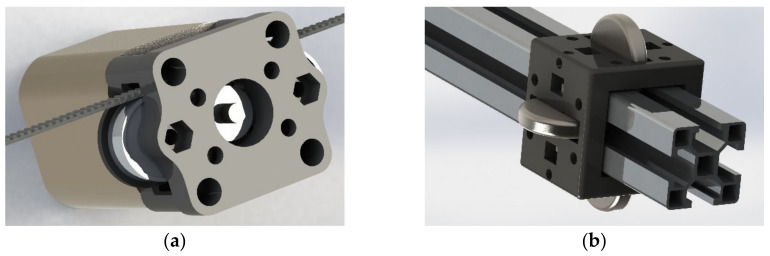
CAD models of components used to construct the three-axis linear robot. (**a**) 3D printed component attached to the face of each stepper motor uses deep groove ball bearings to wrap a timing belt around a timing pulley, coupled with the motor. (**b**) 3D printed linear bearing with fixed axial rotation, steel wheels, and M4 boltholes for integrating components.

A three-axis linear robot was built and used to facilitate the prototyping of the detection system and for testing the position estimation code (see Figure 1). Our proposed method requires approximately eight pictures to be taken with the camera facing the same direction, making the scanning process far simpler and less computationally expensive than full plant-scan based methods, such as the one seen in [22]. In addition, the vertical probing arm provides a minimally invasive method for leaf phenotyping over the robot’s full horizontal reach and acts as a mock end effector where an additional sensor or system could be attached. Axes were positioned using belt drives driven by NEMA 17 stepper motors. Turned steel wheels, held in place with 3D printed PLA blocks (see Figure 2), acted as linear bearings, and allowed motion along 30 mm × 30 mm aluminium profile extrusions with 8 mm slots, which formed the robot’s main frame. This allowed the autonomous scanning of a 250 mm × 250 mm × 330 mm volume, which could be extended with additional aluminium profile extrusion. The PlayStation Eye camera was mounted at the end of the horizontal arm with a 3D printed mount (see Figure 1) and was used for scanning subject plants. This camera was chosen because its resolution was high enough for a neural network (640 × 480), and it could be connected to the Raspberry Pi (the robot’s main computer) over a USB cable.

### 2.2. Electrical Build Description

**Figure 3:**
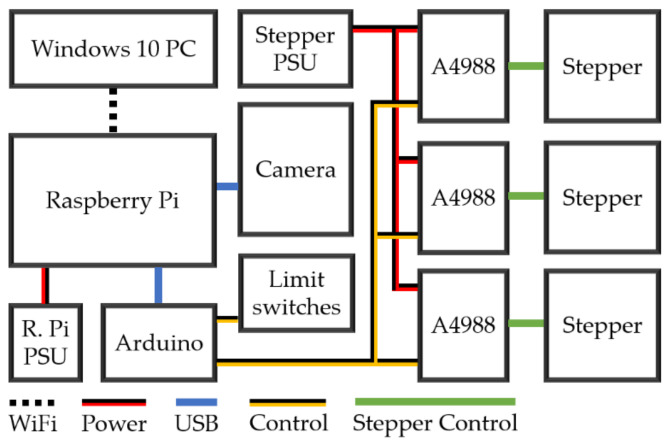
The electronic architecture used for the three-axis linear robot. The Arduino and A4988 motor drivers were soldered onto the same PCB, with the motors, limit switches, Raspberry Pi and power supply connected via header pins, DC connectors and USB.

Requester/responder controller architecture was used with a Raspberry Pi 4 Model B 1 GB (Farnell, Leeds, UK) as the requester, and an Arduino Nano V3.0 ATmega328P (Farnell, Leeds, UK) as the responder. The electronic architecture can be seen in Figure 3. The Raspberry Pi executed the neural network and position estimation code. Commands were sent to the Arduino over the serial port (using a USB cable), which executed movement commands through the control of three NEMA 17 stepper motors via A4988 motor drivers, powered with an external 12 Volt 4 Amp power supply. The Raspberry Pi was controlled remotely over a Secure Shell Protocol and Windows Remote Desktop; however, all plant position estimation computation was performed on the Raspberry Pi to aid in the prototype’s portability, while also minimising development costs. Limit switches connected to the Arduino indicated if any axis had reached the end of its travel.

### 2.3. Software Build Description

**Figure 4:**
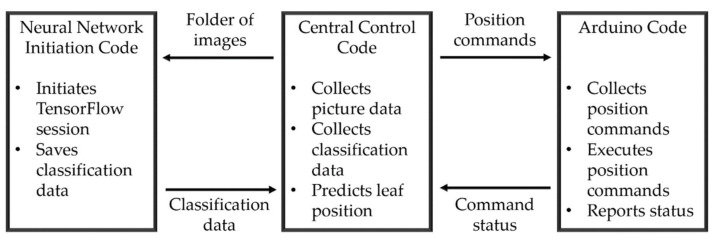
The software architecture used to collect the data required to make plant leaf position estimations. The neural network initiation code was called from the central control code by importing it as a library. Using the command status communications from the Arduino, the central control code could know the current position of the camera and probing arm, and thus know when to take a picture or initiate a chemical measurement.

The software design had three sections: a central control code, the neural network initiation code and Arduino motor control code. The software architecture can be seen in Figure 4. The central control code was run on the Raspberry Pi and called on the neural network initiation code with an image directory parameter. Images in the directory were then run through the neural network and bounding box output data were saved in a known location and format. The Arduino code communicated with the central control code over a serial interface. The central control code transmitted positional commands to the Arduino, which returned signals to indicate if a command had failed, been received or was completed.

#### 2.3.1. Neural Network Training and Initiating

**Figure 5:**
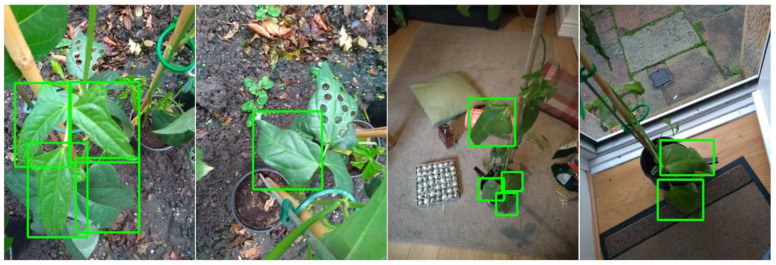
Four example pictures used for training the neural network. Leaves were only selected if they were not occluded or cropped—the method for estimating position is reliant on full leaves being labelled by the classifier.

A dataset of 182 Cowpea plant images (with a total of 361 labelled leaves) was taken using a smartphone camera. The pictures were cropped and scaled-down from 3456 × 4608 to 640 × 480 to match the resolution of the PlayStation Eye Camera, and clear and large leaves were then labelled with bounding boxes. Four example pictures used for training are shown in Figure 5. Light and dark areas, as well as arbitrary objects, were used as the background of each picture to improve the network’s resilience to new scenarios.

The TensorFlow Object Detection API was used with TensorFlow’s deep learning library (version 1.13.2) [24], which is an opensource platform for machine learning. A Windows 10 PC with a Nvidia GeForce GTX 1060 3 GB was used for training and the Raspberry Pi 4 Model B 1 GB for testing. Using the API and utility code from [25], the cowpea dataset was transfer-learned onto the object detection neural network “ssd_mobilenet_v2_quantized_coco”, from the TensorFlow Model Zoo [26]. The batch size was set to three, and the training process underwent 90,000 steps, which took around 20 h. Detections were only accepted if they had a confidence of over 60%. We found this network to be the most accurate which could run on the Raspberry Pi. More computationally intensive models, such as ones based on “Faster RCNN” and “R-FCN” architectures, were found to run into out-of-memory related issues. “ssd_mobilenet_v2_quantized_coco” was also tested using TensorFlow-lite, a deep learning library designed to run on low power machines, however, this drastically decreased the accuracy of the network.

#### 2.3.2. Leaf Detection Grouping

**Figure 6:**
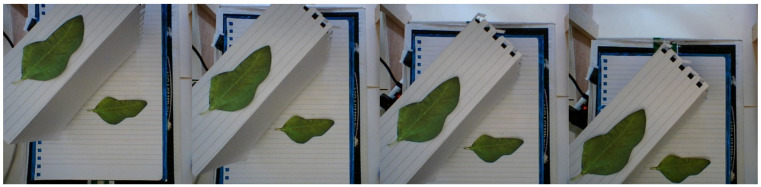
A sample of the pictures taken by the camera that will be used for leaf position estimation. The spacing between each consecutive picture is constant, and the camera is moved in a straight line. Initial tests involved manually positioned printed leaves so the maximum expected accuracy with the current method could be determined.

To predict the location of leaves, a plant is first placed under the three-axis linear robot. The Raspberry Pi sends position commands to the Arduino, which controls the motion of the stepper motors, and moves the camera to specific positions looking down on the plant. At each point, a picture is taken and saved. Six pictures were taken initially, with each position forming a straight line crossing the centre of the robot’s horizontal reach. Four of these pictures can be seen in Figure 6. Positions on the line have equal spacing.

Every picture taken is run though the trained object detection neural network and the detected leaf positions saved. A custom-designed unsupervised grouping algorithm is run on the detection data to group detections of the same leaf. The algorithm creates an object for each detection, with the properties: bounding box size and position; image ID; detection ID; leaf ID. The last is initially undefined, and the algorithm attempts to define it.

**Figure 7:**
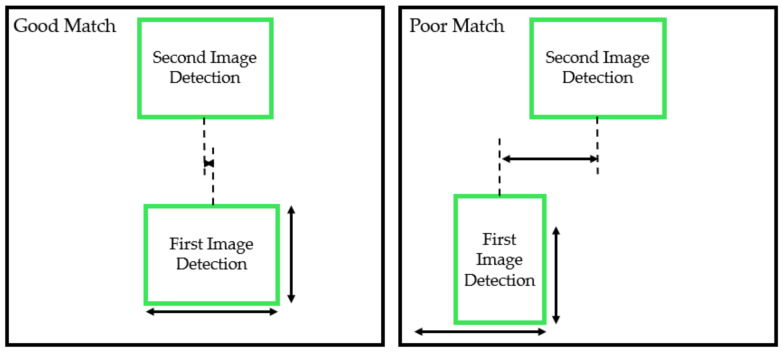
Visual representation of how detections in consecutive images are compared. If the two points are similar enough, the second detection is given the same class as the first. As the camera is moved in a straight line, leaves in the camera’s view should not greatly move from side to side from the camera’s perspective, only up and down, which can be used to predict depth.

The algorithm initially looks at all detections in the first image and gives each detection a unique leaf ID because the same leaf cannot exist in the same image more than once. The algorithm subsequently iterates through each detection in the second image, and for each detection, iterates through each detection in the first image. For each pair of detections, a match probability is defined with Equation (1):

(1) $$P=\frac{1}{k_{s}B_{s}+k_{p}B_{p}}$$

where $B_{s}$ is the summation of the absolute differences between the first and second detection’s width and height, and $B_{p}$ is the absolute difference in x position between the first and second detection’s centre position. $k_{s}$ and $k_{p}$ are constant gains which are used to control the proportional effect $B_{s}$ and $B_{p}$ have on the probability P. By comparing the probability between a point in the second image, and all points in the previous image, the best matching detection can be found for the detection in the second image. Figure 7 shows a visual representation of both a good and poor match. If the probability is above a defined threshold, then the detection in the second image is given the same leaf ID as the matching one in the first image. If the threshold is not passed, then the detection in the second image is assigned a new leaf ID.

**Figure 8:**
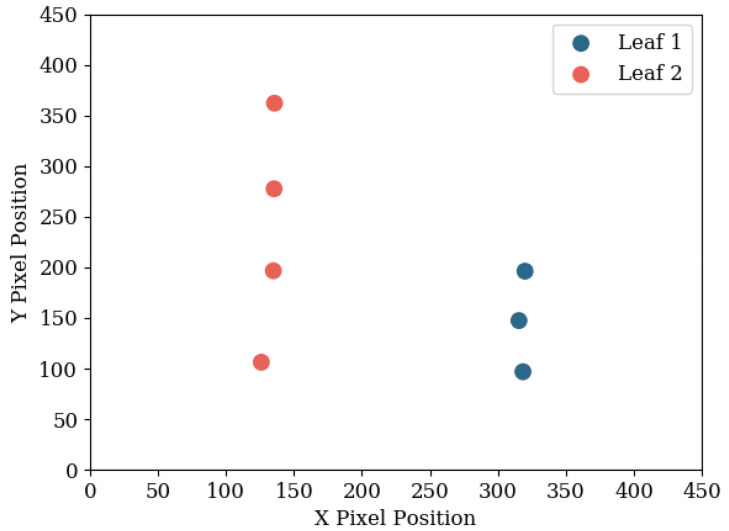
Detections from the images in Figure 6 which have been grouped according to the origin leaf. The linearity of the camera’s movement causes the separation between detection to be almost linear. As the detections of Leaf 2 are more spread out, the leaf is known to be located closer to the camera.

After a comparison has been made between all detections in the first and second image, the algorithm repeats the comparison process in the second and third image, and so on until all images have been compared and assigned a leaf ID. A check is also performed to make sure two detections in an image are not given the same leaf ID, because they both share similar shape and position. In this scenario, previous detections are used to judge which detection is correct, and the incorrect detection is given a new leaf ID. As the algorithm only links up detections from consecutive images, a post-processing operation is performed to group together detections when a leaf detection is missed. This operation also removes outlying datapoints that were misclassified. Figure 8 shows the overlaid detection centre points of the leaves in Figure 6, which were run through the neural network and the unsupervised grouping algorithm.

**Figure 9:**
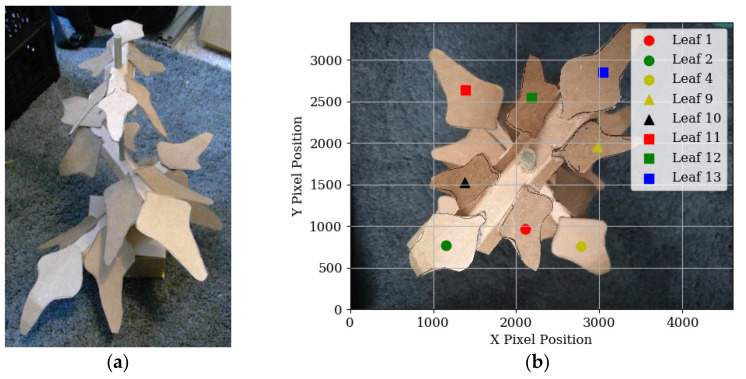
(**a**) Artificial plant used to test the capabilities of the leaf detection grouping algorithm. The plant was photographed in the same manner as the leaves in Figure 6 and labelled manually because the neural network was not trained to detect the fake leaves. (**b**) shows the centre position of each manual detection of one of the photographs.

**Figure 10:**
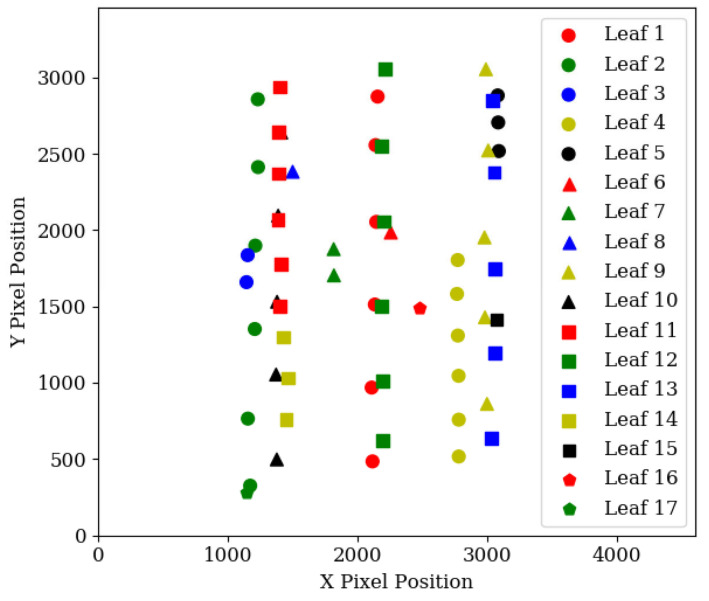
Bounding box data from the artificial plant that have been grouped according to origin leaf, using the unsupervised grouping algorithm. The algorithm successfully grouped all instances of the same leaf.

The algorithm was also tested with edge cases—an artificial plant (Figure 9a) with a high leaf density was created, and all visible leaves manually labelled. An example scanning picture can be seen in Figure 9b. The bounding box data was sent to the algorithm for processing, and the output can be seen in Figure 10.

Although this test did not make use of data from the neural network, it demonstrates the capability of the grouping algorithm to match leaves of varying sizes and heights. Using only nine images, sufficient data were gathered to predict the position of 13 of the 18 leaves, which ranged in length from 55 mm to 115 mm, and in height from 90 mm to 340 mm.

#### 2.3.3. Depth and Position Estimation

**Figure 11:**
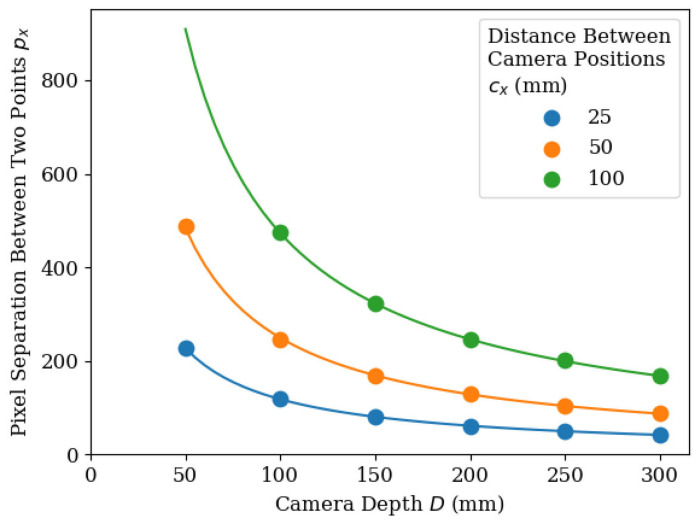
Graph showing the relationship between camera depth and pixel separation between two points. Two points were set apart at distances of 25 mm, 50 mm and 100 mm. The camera was set to face the points at known depths and the pictures were taken. The pixel separation between the points in each image was plotted.

The distance between consecutively detected centre points of the same leaf was used to predict the depth of each leaf. Tests with the PlayStation Eye Camera (the camera used for scanning the plant) were performed to find this relationship. Figure 11 shows the relationship between pixel separation of two points separated by 25 mm, 50 mm, and 100 mm, and the cameras depth.

A power relationship was found between point separation and depth. This is shown in Equation (2), where D is an object’s depth from the camera, $c_{x}$ is the distance between the two positions in which the camera took a picture and $p_{x}$ is the pixel distance between objects in each image. Using this method for calculating depth relies on the assumption that the leaf is parallel to the camera’s CCD circuit. While this may not be an issue when scanning smaller leaves, it could potentially negatively affect the estimation when scanning larger leaves. This could be addressed with additional post-processing, as outlined in Section 4.2.

(2) $$D=\sqrt[{-0.95}]{\frac{p_{x}}{381c_{x}}}$$

**Figure 12:**
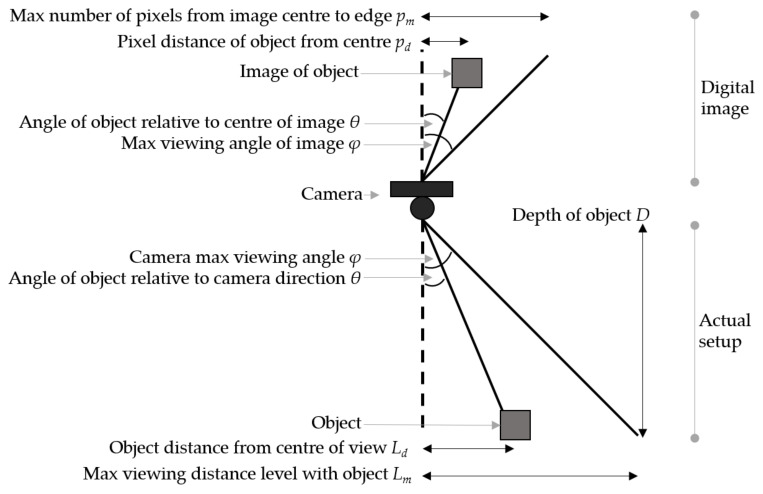
Diagram showing how the 3D position of an object can be calculated from the object’s depth, and its relative pixel position in an image. The angle of the object from the camera’s centre view is equivalent to the pixel angle of the object in the image. After performing two calculations for both the object’s width and height, its position can be estimated.

Knowing the depth, the relative horizontal position can be calculated using trigonometric identities. Figure 12 shows how this was derived.

The distance in millimetres from the centre of a camera’s view to a located object was defined, as shown in Equation (3). $p_{d}$ is the pixel distance between the centre of the image and the object, φ is the max viewing angle of the camera, and $p_{m}$ is the number of pixels between the edge and centre of the image.

(3) $$L_{d}=D\tan\left(sin^{-1}\left(\frac{p_{d}\sin\left(\varphi \right)}{p_{m}}\right)\right)$$

**Figure 13:**
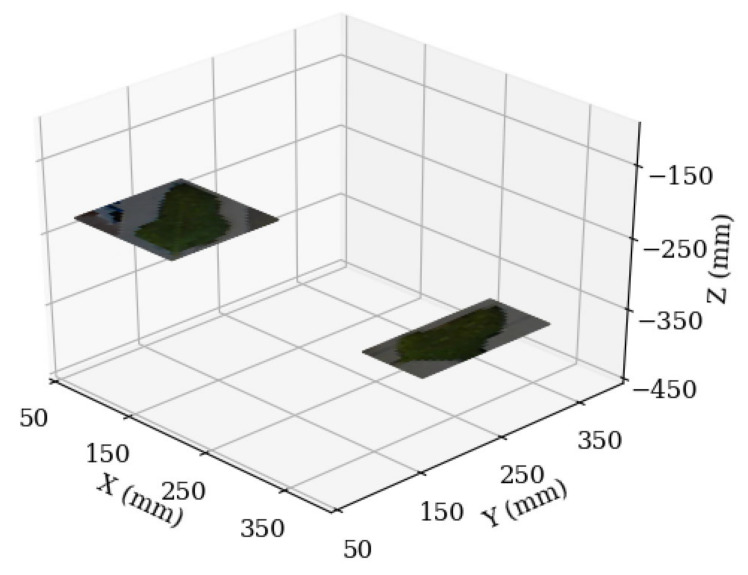
3D digital reconstruction of the leaves seen in Figure 6. Creating this graph demonstrates that the functionality of leaf position estimation could run from end to end with no intervention.

Using Equations (2) and (3), the positions of the leaves detected in Figure 6 were estimated. By cropping the image down to the detected leaves, and scaling each detection based on its depth, a 3D digital reconstruction (Figure 13) was created. A GitHub link providing the code for the motor control and image analysis can be found in the Supplementary Materials.

## 3. Results and Discussion

Forty-two images with a total of 106 visible leaves were run through the neural network on the Raspberry Pi. The neural network successfully detected 54 leaves (51% accuracy). A key point to note is that the 42 images were taken from a set of 6 plants, so there were multiple duplicate leaves. It was observed that if a leaf was detected in an image, then the probability of the same leaf being detected in a different image was 69%. This is beneficial for the proposed grouping algorithm which relies on consecutive detections. Unclear leaves, which may be more unsuitable for phenotyping, were never detected.

**Figure 14:**
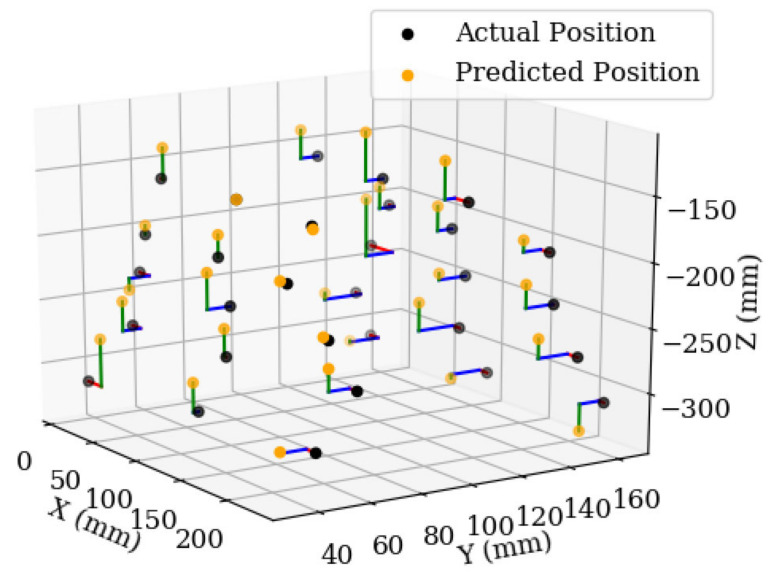
Actual and estimated position of the 30 test leaves. Each leaf was positioned to test the full range of the robot, to see if there was a bias in any direction.

Printed paper leaves were placed in 30 different positions in groups of three and the process was run end to end. The estimated position of each leaf was compared to the actual position (Figure 14). The purpose of this test was to determine the highest accuracy expected of the proposed method, including the accuracy of the neural network, and the accuracy of the derived depth and position estimation techniques, and to observe whether accuracy was affected by actual leaf position. Due to this, the same printed paper leaf was used in every position.

The average of the total error of every measurement was 20 mm and the maximum total error of a measurement was 52 mm. Artificial leaves were placed between depths of 150 mm and 300 mm from the camera—although the robot had a total reachable area of 250 mm × 250 mm × 330 mm, a leaf could only have its position estimated when at least two images were taken of the entire leaf. If the leaf is partially outside of the cameras field of view, then the centre position will not be accurate. Mounting the camera at a higher position would improve this, but also decrease accuracy. Figure 14 shows that most measurements had a depth measurement error, and because the x and y positions were calculated from the depth, an inaccurate depth measurement led to inaccurate horizontal positions. By rearranging Equation (2), making $p_{x}$ the subject, the expected pixel error of the neural networks detections which would cause an error of 20 mm was calculated at depths of −150 and −300 mm. At −150 mm, it would take an error of 15 pixels for 20 mm of depth error. At −300 mm, it would take an error of 4 pixels for a 20 mm of depth error. This suggests that an increase in error at lower depths was to be expected.

**Figure 15:**
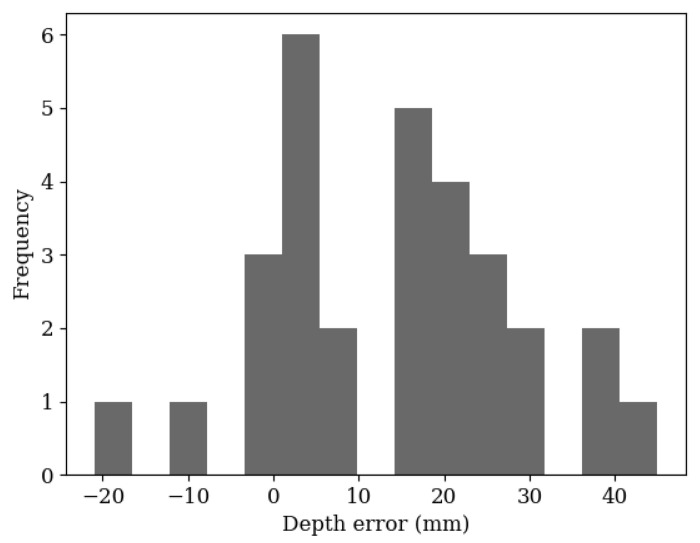
A histogram plot of the depth error in 30 leaf position estimations. The data shows a positively skewed bell curve, centred around 5 mm. The RMS error is 20 mm, ranging between −20 mm and 40 mm.

Figure 15 shows that although there is a slight systematic error of overestimating the depth, the large spread indicated that the error is due to the input data from the neural network. The error in estimations from the neural network is visible in Figure 8—the centre positions from each detection do not perfectly line up, and they are not equally spaced.

**Figure 16:**
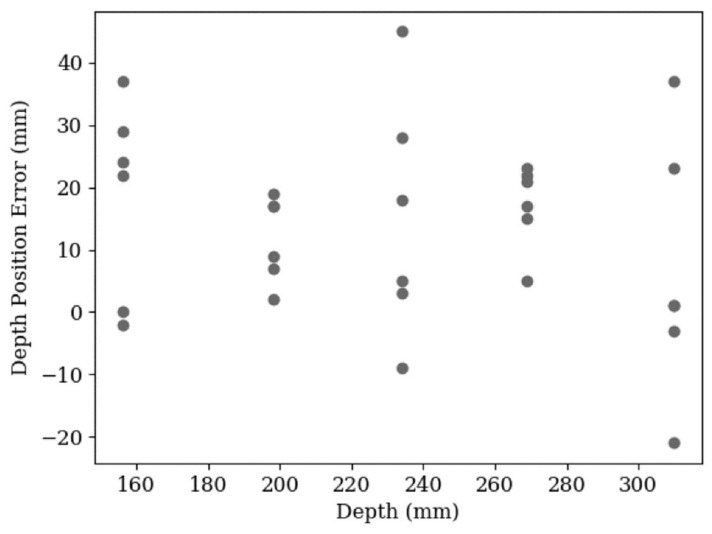
A scatter plot of the depth error in 30 leaf position estimations plotted against depth. The data shows a spread from −21 mm to 45 mm. The RMS error is 20 mm.

Figure 16 shows that there was also no correlation between depth and depth error, which was previously expected.

**Figure 17:**
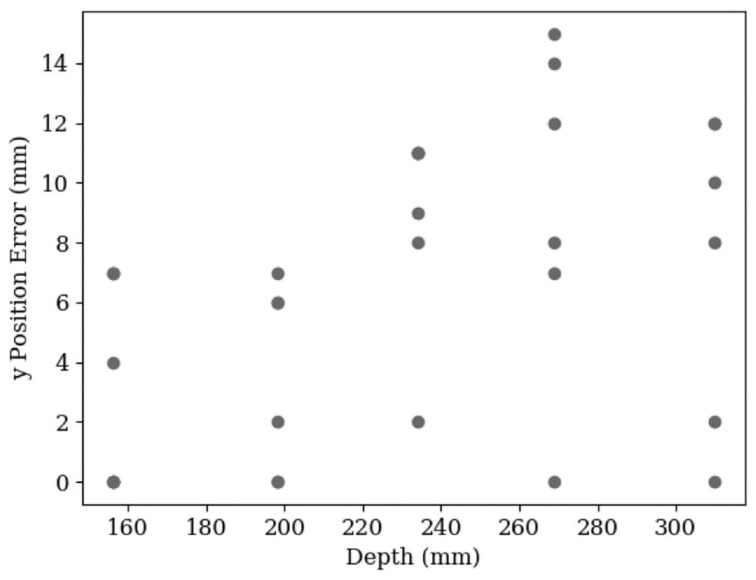
A scatter plot of the y-axis error in 30 leaf position estimations against depth. The data shows a spread from 0 mm to 15 mm. The RMS error is 8 mm.

Compared to the depth error, Figure 17 shows the y-position error has less variation. An increase in the spread of results was expected at higher depths because of the smaller pixel separation between points, but the data doesn’t show a strong indication of this, suggesting again that the primary source of error was due to the unpredictability of the neural network. There is also a systematic error apparent from the fact that no y-position was underestimated. This error was due to an inaccurate measurement between the camera lens and the probing arm and could be corrected with more accurate calibration.

**Figure 18:**
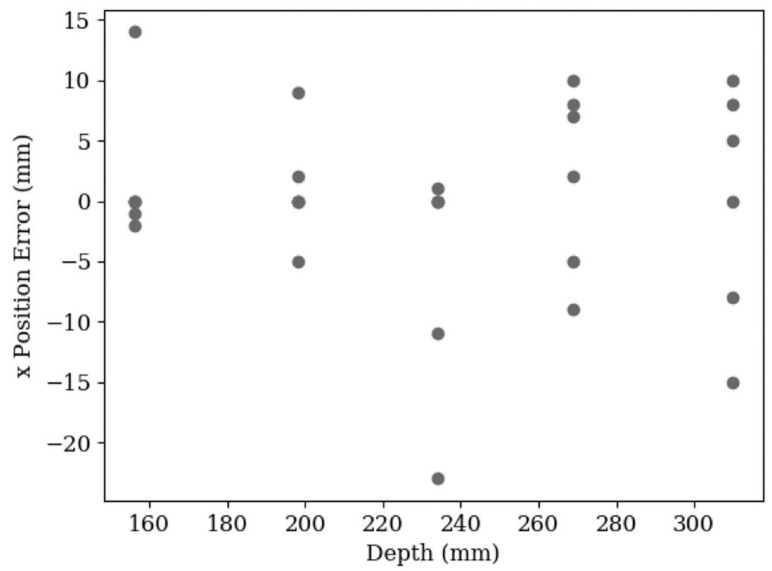
A scatter plot of the x-axis error in 30 leaf position estimations. The data shows a spread from −23 mm to 15 mm. The RMS error is 8 mm.

It was again expected that there would be an increase in x-position error spread with an increase in depth, however, Figure 18 shows there is little to no indication of this. The randomness of results again points to the conclusion that the error introduced by the neural network is the most prominent.

## 4. Conclusions

A new method for predicting the position of wide leaves has been developed and evaluated. The method is relatively straightforward, hence can be developed and implemented on most systems and furthermore, it is not sensitive to the packing density of the vegetation or natural light. The method also does not rely on vast numbers of input images from multiple angles, or substantial image processing when compared to other image-based object modelling techniques, making it considerably less time and computationally-intensive. By moving a camera over the plant and taking approximately eight pictures, partially obstructed leaves in a canopy are viewable at different angles, and thus their positions are estimated. This capability is demonstrated in Figure 9 and Figure 10 with an artificial plant—lower leaves which were obstructed by higher leaves in Figure 9b were able to be measured from other scanning pictures. Plants with a particularly close canopy structure, such that some lower leaves always remain obstructed during the scan, would presumably not be suitable for autonomous scanning, as they would likely be unreachable without a riskier invasive manoeuvre.

We have identified several clear areas for development, which could rapidly improve results and make the method suitable for application on live plants. These are described below and will be attempted in future projects with a wider scope.

### 4.1. Neural Network

The data above indicates that the primary reason for inaccurate measurements was due to the accuracy of the neural network—the proposed bounding boxes did not always precisely wrap around any detected leaf. This can be seen from the almost linearly spaced points in Figure 8. A solution to this would be to implement a more powerful network. “ssd_mobilenet_v2_quantized_coco” is intended to run on low-power computers, at a cost of accuracy. Through testing, it was found that it was the most accurate network capable of running on the 1 GB version of the Raspberry Pi 4 Model B. An 8 GB model is also available, which may allow a more accurate network to be implemented. Another potential solution would be the addition of a Coral USB Accelerator with the Raspberry Pi or the use of a Nvidia Jetson Nano as the primary computer. If computer networking is available, then a backend server with a powerful GPU could perform the detections, allowing a more accurate network to be implemented. Faster detections would also permit more scanning photos of the plant to be taken, giving the leaf position estimation code more data to work with, further improving accuracy.

If more computing power is not available, an alternative solution would be to use a higher resolution camera. The camera used had a resolution of 640 × 480, which was small enough to be processed in the neural network on the Raspberry Pi. An alternative approach would be to have multiple detection stages. This would involve taking pictures with a high-resolution camera, downsizing them so they can be processed to find the approximate location of any leaves, then cropping the high-resolution images down to each leaf’s approximate location. Finally, the cropped high-resolution image could be run through the network to obtain a more accurate result.

### 4.2. Proposed Position Estimation Technique

The main limiting factor of the proposed method is the assumption that every leaf’s surface is parallel with the camera’s CCD circuit—if a leaf is not perfectly flat, then the bounding box shape in consecutive pictures will vary, affecting the leaf’s estimated position. This issue could be solved with a post processing task after a leaf has been identified, located and isolated. The task would involve the identification of common visible points on a leaf’s surface, such as the tip, base, and vein structure, where again a similar grouping algorithm and position estimation calculations could be applied to determine the rotation and shape of the leaf. This would require a slightly higher identification accuracy than the presented neural network but could be achieved with additional computational resources.

The unsupervised detection grouping algorithm proved successful with the test data put through it, however, there is scope for further advances applying the algorithm to group detections that are not in consecutive images. Preliminary research was performed into this but all proposed algorithms had very high order time complexity and were therefore unsuitable for making quick position estimations.

As the position estimation technique is based on geometric equations, most uncertainties in the algorithm were systematic and could be addressed with variable fine-tuning and scaling. An example of this would be the use of an image scaling calibration step to remove inaccurate detections close to the edge of images if the camera used for testing had strong fish-eye distortions.

## Supplementary Materials

Code written for this article, all data collected, and the trained neural network can be accessed in this GitHub Repository to enable our experiments to be reproduced: https://github.com/JamAJB/Plant-Leaf-Position-Estimation-with-Computer-Vision.
